# Antibiotic resistance patterns of bacteria causing urinary tract infections in the elderly living in nursing homes versus the elderly living at home: an observational study

**DOI:** 10.1186/s12877-015-0097-x

**Published:** 2015-08-04

**Authors:** Mark Fagan, Morten Lindbæk, Nils Grude, Harald Reiso, Maria Romøren, Dagfinn Skaare, Dag Berild

**Affiliations:** Institute of Health and Society, Faculty of Medicine, University of Oslo, Postboks 1130, Blindern, 0318, Oslo Norway; Antibiotic Centre for Primary Care, University of Oslo, Postboks 1130, Blindern, 0318, Oslo Norway; Department of Microbiology, Vestfold Hospital Trust, Tønsberg, Postboks 2168, 3103 Tønsberg, Norway; Department of Infectious Disease, Institute of Clinical Medicine, Faculty of Medicine, University of Oslo, PO Box 4950, Nydalen, 0424, Oslo Norway

**Keywords:** Urinary tract infection, Antibiotic resistance, Antibiotic stewardship, Nursing home, Geriatrics, Guidelines

## Abstract

**Background:**

Antibiotic resistance is a problem in nursing homes. Presumed urinary tract infections (UTI) are the most common infection. This study examines urine culture results from elderly patients to see if specific guidelines based on gender or whether the patient resides in a nursing home (NH) are warranted.

**Methods:**

This is a cross sectional observation study comparing urine cultures from NH patients with urine cultures from patients in the same age group living in the community.

**Results:**

There were 232 positive urine cultures in the NH group and 3554 in the community group. *Escherichia coli* was isolated in 145 urines in the NH group (64 %) and 2275 (64 %) in the community group. There were no clinically significant differences in resistance.

Combined, there were 3016 positive urine cultures from females and 770 from males. *Escherichia coli* was significantly more common in females 2120 (70 %) than in males 303 (39 %)(*p* < 0.05). *Enterococcus faecalis* was significantly less common in females 223(7 %) than males 137 (18 %) (*p* < 0.05). For females, there were lower resistance rates to ciprofloxacin among *Escherichia coli* (7 % vs 12 %; *p* < 0.05) and to mecillinam among *Proteus mirabilis* (3 % vs 12 %; *p* < 0.05).

**Conclusions:**

Differences in resistance rates for patients in the nursing home do not warrant separate recommendations for empiric antibiotic therapy, but recommendations based on gender seem warranted.

## Background

Antibiotic resistance is a worldwide problem threatening our ability to treat infections [1]. Infections caused by multi-resistant bacteria are increasing among the elderly living in nursing homes (NH) [2–4]. The elderly are the age group with the highest prevalence of antibiotic use in Norway [5]. Anatomical and physiologic changes caused by aging [6, 7], usage of urinary catheters, nasogastric and percutaneous feeding tubes and intravenous catheters are common in NH, all predisposing to bacterial colonization and infections [8].

Urinary tract infections (UTI) are the most common infections among NH patients [9, 10]. However, a considerable proportion of antibiotic prescribing for presumed UTI is questionable. Treatment of asymptomatic bacteriuria (ASB), and non-specific symptoms inaccurately interpreted to be caused by UTIs is prevalent in the NH despite no evidence of benefit and guidelines dissuading this practice [11–14]. Inappropriate antibiotic use is an important factor contributing to antibiotic resistance [1]. It is therefore necessary to optimize antibiotic prescribing for UTI in the institutionalized elderly. To accomplish this, knowledge of antibiotic resistance in the NH is essential.

Studies from abroad indicate that the bacterial etiology and resistance rates of UTI in NH patients resemble hospitalized patients more than they do the elderly living in the community [15, 16]. Results from a recent Australian study suggests that guidelines for empiric treatment of elderly patients with UTI should take into consideration differences in antibiotic resistance patterns in bacteria causing infections in the elderly living in NH versus the elderly living at home [17]. However, antibiotic resistance varies greatly between countries. Whether these findings are relevant in Norway or other countries is unknown.

Several articles address differences in the prevalence and microbiologic etiology of UTI in elderly women or men, but separate therapy suggestions based on gender are not specified [10, 18]. In addition, guidelines often lack specific recommendations based on gender in the elderly [19, 20].

We aim to assess whether the difference in resistance rates of bacteria isolated from NH patients compared to community dwelling elderly is so great that separate recommendations for empiric antibiotic therapy for UTI for the two groups is necessary. Second, we aim to assess if gender specific recommendations for antibiotic therapy of UTI are warranted in the elderly.

## Methods

### Setting/patients

The laboratory for medical microbiology, Vestfold Hospital Trust serves the population of Vestfold County (240,000 inhabitants) [21]. NH patients resided in 34 different NHs in Vestfold County, were 65 years and older with a positive urine culture in the time period from 16. Nov 2009 through 31. Des 2010 (NH group). Results were compared to all positive urine cultures from non-hospitalized patients in Vestfold County 65 years or older not living at a NH in the same time period, the community dwelling group (CD). We registered both microbes when two microbes with significant bacteriuria were present.

### Study design

The study is a cross-sectional observational study. The microbiologic laboratory registers all urines received for analysis and enters the patient’s full name, person number (unique for each person in Norway), address, the ordering physician and the institution (nursing home, hospital, private practice, emergency call service, visiting nurse service). In the CD group we excluded hospitalized patients, urines ordered by the visiting nurse service and urines taken in the emergency room.

### Microbiologic analysis and susceptibility testing

Urine cultures fulfilling the criteria for significant bacteriuria (≥10,000 colony-forming units/mL urine) were included in the study. For Gram negative rods the automated method Vitek 2 (bioMérieux) was used. Appropriate antibiotics were selected for each bacterial species according to recommendations from the Norwegian Working Group on Antibiotics (NWGA) [22]. Results were interpreted according to clinical breakpoints from NWGA which are based on those from The European Committee on Antimicrobial Susceptibility Testing EUCAST [23]. Dip-agar isolates and Gram positive bacteria were tested by disk diffusion technique (Oxoid) after the manufacturer’s recommendations. Resistance values were recorded either as susceptible (S), intermediate (I), or resistant (R).

Resistance data were extracted from the laboratory’s database.

### Norwegian guidelines for empiric therapy for UTI in the nursing home [19]

ᅟ

Lower UTI: Pivmecillinam, trimethoprim, nitrofurantoin.

Upper UTI: Pivmecillinam, trimethoprim-sulfamethoxazole, ciprofloxacin, ofloxacin.

### Outcomes

1. Frequency and susceptibility of bacteria cultured in urine specimens.
2. Susceptibility for each of the five most common uropathogens: *Escherichia coli* (*E coli*), *Klebsiella pneumoniae (K pneumoniae*), *Proteus mirabilis (P mirabilis)*, *Enterococcus faecalis (E faecalis)*, and *Pseudomonas aeruginosa (P aeruginosa)*.

### Calculating the theoretic risk of therapy failure

The theoretic risk of antibiotic failure depends on two factors; the prevalence of the microbes in the patient population and the resistance rate of these microbes to the chosen antibiotic [24]. We calculated the theoretical risk of therapy failure by multiplying the relative percentage each microbe was responsible for (e.g. for *E coli* 70.3 % for females and 39.4 % for males) with the resistance rate for that antibiotic (e.g. ciprofloxacin for *E col*; 7.2 % for females and 11.9 % for males). We performed these calculations for each of the five most commonly isolated microbes; *E coli, E faecalis, K pneumoniae, P mirabilis, and P aeruginosa*.

### Statistical analysis

IBM SPSS® statistics program was used for statistical analysis. We used a significance level of *p* < 0.05.

We used Pearson Chi^2^ test to compare differences in gender distribution between the NH group and the CD group and the *t*-test for independent samples to compare the mean age in the two groups.

We used the Pearson Chi^2^ test and the Fischer’s exact test (when appropriate) to compare differences in resistance rates for relevant antibiotics between the NH group and the CD group, and between males and females.

### Ethical considerations

The study has been approved by the Norwegian regional ethics committee. The ethics committee waived the requirement for informed consent for this study. (REK sør-øst 2013/2282)

## Results

There were 3786 bacteria isolates, 232 from NH patients and 3554 from CD patients. Females contributed 183 (78.9 %) and 2833 (79.7 %) and males 49 (21.1 %) and 721 (20.3 %) of the isolates to the NH and CD groups respectively (*p* =0.76).

The mean age of females in the NH group was 87.0 (SD 6.7; 95 % CI, 86.0-88.0) compared to 79.3 (SD 8.1; 95 % CI, 79.0-79.6) in the CD (*p* < 0.05). The mean age for males in the NH group was 83.1 (SD 6.2; 95 % CI, 81.3-84.9) compared to 78.0 (SD 7.6; 95 % CI, 77.8-78.9) in the CD group (*p* <0.05).

### NH vs CD

There was no significant difference in the proportions of any of the bacteria between patients in the NH group and the CD group as a whole (Table 1). *E coli* was the most common and *E faecalis* the next most common pathogen isolated in both groups. In the category “Other bacteria”, no single bacterium contributed more than 2.5 % to the total in either the NH or the CD group.

**Table 1:** Urine culture results from patients living in a nursing home (NH) compared to community dwelling elderly (CD) in Vestfold County, Norway 2010

			*E coli*	*E faecalis*	*K pneumoniae*	*P mirabilis*	*P aeruginosa*	*Other* ^*a*^
NH	Female	n (%)	133 (72.7)	11 (6.0)	5 (2.7)	8 (4.4)	4 (2.2)	22 (12.0)
	Male	n (%)	15 (30.6)	8 (16.3)	6 (12.2)	4 (8.2)	2 (4.1)	14 (28.6)
		p-value	< 0.01^b^	0.47	0.28	0.61	0.55	0.21
CD	Female	n (%)	1987 (70.1)	212 (7.5)	165 (5.8)	93 (3.3)	32 (1.1)	344 (12.1)
	Male	n (%)	288 (39.9)	129 (17.9)	43 (6.0)	38 (5.3)	25 (3.5)	198 (27.5)
		p-value	< 0.01^c^	< 0.01^c^	0.96	0.59	0.54	< 0.01^c^
	NH	n (%)	148 (63.8)	19 (8.2)	11 (4.7)	12 (5.2)	6 (2.6)	36 (15.5)
	CD	n (%)	2275 (64.0)	341 (9.6)	208 (5.9)	131 (3.7)	57 (1.6)	542 (15.3)
	Total	n	2423	360	219	143	63	578
		p-value	0.48	0.42	0.43	0.6	0.86	0.49

^a^Other was comprised of 18 different microbes in the NH group and 24 different microbes in the CD group

^b^Comparison between females and males in the NH > 65 years old

^c^Comparison between CD females and males > 65 years old

*E coli* resistance rates were over 20 % for both ampicillin and trimethoprim in both groups (Table 2). The NH group showed a slightly though non-significantly higher resistance rate for ciprofloxacin than the CD group (9.5 vs 7.7 %). *E faecalis* resistance rates for trimethoprim were over 20 % in both groups but exhibited no resistance to ampicillin or nitrofurantoin in either group. *K pneumonia*e and *P mirabilis* resistance rates for ciprofloxacin were significantly higher in the NH group (*p* < 0.05).

**Table 2:** Resistance rates of the five most commonly isolated uropathogens from patients living in nursing homes (NH) compared to community dwelling elderly (CD) in Vestfold County, Norway 2010

	*E coli*			*E faecalis* ^*a*^			*K pneumoniae*			*P mirabilis*			*P aeruginosa*		
	NH	CD		NH	CD		NH	CD		NH	CD		NH	CD	
	n (%)	n (%)	p-value	n (%)	n (%)	p-value	n (%)	n (%)	p-value	n (%)	n (%)	p-value	n (%)	n (%)	p-value
Ampicillin^b^	48 (32)	732 (32)	0.95	0	0		Res^c^	Res		2 (17)	14 (11)	0.63	Res	Res	
Ciprofloxacin^d^	14 (10)	175 (8)	0.08	NA^e^	NA^e^		2 (18)	7 (3)	0.07	3 (25)	7 (5)	0.04^h^	0 (0)	5 (9)	0.6
Mecillinam	4 (3)	83 (3.6)	0.82	NA^f^	NA^f^		1 (9)	16 (8)	0.62	1 (8)	6 (5)	0.51	Res	Res	
Nitrofurantoin	3 (2)	39 (2)	0.76	0	0		Res	Res		Res	Res		Res	Res	
Trimethoprim^g^	36 (24)	513 (23)	0.1	6 (32)	93 (27)	0.69	1 (9)	39 (19)	0.46	3 (25)	23 (18)	0.79	Res	Res	

^a^Resistance rate for Vancomycin was 0 % in both groups

^b^For *E coli, and P mirabilis* intermediate (I) is classified as sensitive (S) according to recommendations from Norwegian Working Group on Antibiotics

^c^Res: Resistant

^d^(S) classified as (I) if the microbe in question is resistant (R) for nalidixic acid

^e^NA: not applicable. Minimum inhibitory concentrations are so high that ciprofloxacin is not recommended for infections due to *E Faecalis*

^f^NA: not applicable. Mecillinam is ineffective against *E Faecalis* in vitro

^g^For *E faecalis* intermediate (I) is classified as sensitive (S)

^h^Significant at α = 5 %

### Male vs female (irrespective of residence)

The difference in the relative frequency of which bacteria were isolated between males and females was highly significant (*p* < 0.05) (Table 1). For *E coli* there was a significantly higher resistance rates for ciprofloxacin for males than for females (*p* < 0.05). For *P mirabilis* there was a significantly higher resistance rates for mecillinam for males than for females (*p* < 0.05) (Table 3). There were no significant differences in resistance rates between males and females for *E faecalis*, *K pneumoniae* or *P aeruginosa*.

**Table 3:** Resistance rates of the five most commonly isolated uropathogens from patients 65 years old or older in Vestfold County, Norway 2010: females compared to males (irrespective of residence)

	*E coli*			*E faecalis* ^*a*^			*K pneumoniae*			*P mirabilis*			*P aeruginosa*		
	NH	CD		NH	CD		NH	CD		NH	CD		NH	CD	
	n (%)	n (%)	p-value	n (%)	n (%)	p-value	n (%)	n (%)	p-value	n (%)	n (%)	p-value	n (%)	n (%)	p-value
Ampicillin^b^	680 (32)	100 (33)	0.74	0	0		Res^c^	Res		11 (11)	5 (12)	0.99	Res	Res	
Ciprofloxacin^d^	153 (7)	36 (12)	0.03^h^	NA^e^	NA^e^		5 (3)	4 (8)	0.12	8 (8)	2 (5)	0.72	4 (11)	1 (4)	0.38
Mecillinam	74 (4)	13 (4)	0.51	NA^f^	NA^f^		13 (8)	4 (8)	0.32	2 (2)	5 (12)	0.02^h^	Res	Res	
Nitrofurantoin	39 (2)	3 (1)	0.54	0	0		Res	Res		Res	Res		Res	Res	
Trimethoprim^g^	490 (23)	59 (20)	0.36	65 (29)	34 (25)	0.1	27 (16)	13 (27)	0.1	22 (22)	4 (10)	0.4	Res	Res	

^a^Resistance rate for Vancomycin was 0 % in both groups

^b^For *E coli, and P mirabilis* intermediate (I) is classified as sensitive (S) according to recommendations from Norwegian Working Group on Antibiotics

^c^Res: Resistant

^d^(S) classified as (I) if the microbe in question is resistant (R) for nalidixic acid

^e^NA: not applicable. Minimum inhibitory concentrations are so high that ciprofloxacin is not recommended for infections due to *E Faecalis*

^f^NA: not applicable. Mecillinam is ineffective against *E Faecalis* in vitro

^g^For *E faecalis* intermediate (I) is classified as sensitive (S)

^h^Significant at α = 5 %

### Theoretic risk of empiric therapy failure

For females the risk of failure was greater than twenty percent for ampicillin and trimethoprim. For males the risk of failure was greater than twenty percent for ampicillin, ciprofloxacin and mecillinam (Table 4).

**Table 4:** Theoretic risk of failure with empiric antibiotic therapy (%) due to resistance rates in urine cultures from elderly patients (both NH and CD) in Vestfold County Norway 2010^a^

Empiric antibiotic	Female	Male
Ampicillin	29.7	23.6
Ciprofloxacin	13.0	23.4
Mecillinam	11.5	24.2
Nitrofurantoin	11.4	15.9
Trimethoprim	21.2	17.8

^a^Based on the five most commonly cultured bacteria; *E coli, E faecalis, K pneumoniae, P mirabilis*, and *P aeruginosa*. The percentages are calculated by multiplying the percentage each microbe was responsible for by the resistance rate for that microbe

## Discussion

The differences between bacterial etiology and resistance rates among uropathogens isolated from elderly patients living in a NH and those living in the community were clinically unimportant. There is however a significant and clinically relevant difference between males and females both in terms of bacterial etiology and resistance rates.

Therapy decisions in empiric antibiotic therapy are educated guesses based on the relative prevalence of the microbes causing the infection and the resistance rates of these microbes. Guidelines for empiric antibiotic therapy consider both these factors and the severity of the infection. The sum of potentially inadequate coverage (e.g. mecillinam for *P aeruginosa*) or high resistance rates for a specific antibiotic (e.g. ampicillin for *E coli*) will influence recommendations for empiric therapy. Previous studies of urinary tract isolates from hospitalized patients with pyelonephritis and out-patients with uncomplicated UTI have shown that guidelines may need updating to be in concordance with local susceptibility rates [25–27].

Studies indicate increased bacterial resistance in bacteria causing UTI in the elderly living in health care facilities than in community acquired UTI and is similar to hospital acquired UTI [10, 15]. A recent Australian study suggested changes in empiric therapy recommendations for UTI due to higher resistance rates among Enterobacteriacea (*E coli*, *K pneumoniae* and *P mirabilis*) isolated in urine cultures from NH patients than in community dwelling elderly [17]. Our study also showed a higher rate of resistance for Enterobacteriacea in urine cultures from NH patients but comes to a different conclusion. The non-significantly higher rate of ciprofloxacin resistance in E coli from nursing home patients is far below the 20 % resistance rate normally used to dissuade the choice in empiric treatment for non-serious infections like cystitis. Because *K pneumoniae* and *P mirabilis* contribute minimally to the total number of UTI they do not result in a significantly higher risk of therapy failure in the NH despite the higher rates of resistance in these bacteria. Consequently, differences between culture results from the NH and the elderly living in the community do not warrant separate recommendations for empiric antibiotic therapy for cystitis.

Our results do, however, indicate that separate recommendations may be in order for males and females irrespective of where the patient resides. This is not surprising as the bacterial etiology of UTI differs between males and females, a finding reflected in other studies [10, 28]. Ciprofloxacin and mecillinam may be poor choices for empiric treatment for males due to a higher rate of ciprofloxacin resistance among *E coli* and a relatively high prevalence of *E faecalis* for which neither ciprofloxacin nor mecillinam are recommended. The higher prevalence of *E faecalis* in the elderly male population is a finding seen in other studies also [28, 29]. In addition, there are higher rates of mecillinam resistance among *K pneumoniae* and *P mirabilis* in males vs females. These results should be interpreted cautiously due to the uncertainty of catheter status. In the NH, use of permanent urinary catheters is more prevalent in males than in females [30]. Furthermore, bacteria from patients with urinary catheters have a higher rate of resistant organisms [31–33].

Trimethoprim may be a poor choice for females but acceptable in males due to the higher rate of *E coli* resistance in females than in males. Amoxicillin cannot be recommended for empiric therapy for either gender. Treatment of UTI should also take into consideration that even relatively high doses of trimethoprim or nitrofurantoin will not give high enough serum or tissue levels to result in cure for pyelonephritis or prostatitis infections especially if the infection is caused by *E coli* or other Enterobacteriacea species.

The rates of resistance shown in our study are similar to national surveillance rates in Norway, but are somewhat higher than a recent study of a NH in Sweden raising a question of the external validity of our study [31, 34]. A possible explanation for this is that our isolates were from patients having infections which were to be treated while the Swedish study examined urine from all patients able to provide a urine sample. This could potentially make the contribution of ASB higher in the Swedish study.

The concept of external validity is relevant when it comes to studies dealing with empiric antibiotic treatment because resistance problems vary substantially throughout the world. The theoretic model presented in Table 4 is based on local resistance patterns making the issue of its external validity salient. While the results here do not have relevance in other countries, the theoretic basis behind determining the risk of treatment failure is relevant anywhere by using local resistance data [24].

Earlier studies from Norway show lower resistance rates than in this study [35]. This change over time and the differences between the Swedish, Australian and our study illustrate the need for periodic national monitoring of resistance development to keep guideline recommendations up to date and reliable. Reliable guidelines are an important part of antibiotic stewardship but there are numerous barriers impairing adherence to guidelines [36, 37]. Lack of periodic surveillance can undermine the credibility of empiric therapy guidelines, and worse, result in therapy failure for patients.

There are several weaknesses in our study. We are uncertain about the compliance with the Norwegian guidelines outlining correct urine sampling technique in the two groups [38]. Urinary incontinence makes correct urine sampling challenging and is more prevalent in the NH than in the community [39]. Urinary catheter status was not reported systematically in either the NH group or the CD group making conclusions about the contribution of catheter associated infections impossible. Rates of inappropriate collection techniques and the prevalence of cultures from catheterized patients may be different in the two groups. Given this potential selection bias one might expect a higher rate of resistance and higher prevalence of pathogenic bacteria in the NH, but the results in our study show no such difference.

In the NH group, all culture results were from patients with suspected UTI treated with antibiotics. None of the NH urines were due to post-therapy control. Norwegian guidelines dissuade urine culturing for ASB and do not recommend routine post-therapy control [19]. Despite this, the CD group may be less homogeneous because we cannot ascertain the proportion of the culture results due to UTI, ASB or the contribution of post-therapy control cultures. In addition, the relative contribution of lower UTI and upper UTI in the two groups is not precisely known. As the incidence of dementia, use of catheters and urinary incontinence are higher in the NH setting one would expect the rate of complicated UVI inclusive pyelonephritis to be higher in this group [40].

Patients in the NH group were on average 10 years older than the CDs. Antibiotic resistance among uropathogens in general and for ciprofloxacin specifically is higher in the elderly [16, 40–42]. In this case having a younger CD group would contribute to making the difference in resistance rates between the two groups more obvious. That was not the case in this study.

## Conclusion

In Norway, differences in resistance rates for patients 65 years and older living at home or in a nursing home do not warrant separate recommendations for empiric antibiotic therapy but recommendations based on gender seem warranted.

## Abbreviations

ASB: Asymptomatic bacteriuria
CD: Community dwelling
CI: Confidence interval
*E coli*: *Escherichia coli*
*E faecalis*: *Enterococcus faecalis*
EUCAST: European Committee on Antimicrobial Susceptibility Testing
*K pneumoniae*: *Klebsiella pneumoniae*
MIC: Minimum inhibitory concentration
NA: Not applicable
NH: Nursing home
NWGA: Norwegian Working Group on Antibiotics
*P aeruginosa*: *Pseudomonas aeruginosa*
*P mirabilis*: *Proteus mirabilis*
Res: 100 % resistant
UTI: Urinary tract infection
